# Quantifying the barrier lowering of ZnO Schottky nanodevices under UV light

**DOI:** 10.1038/srep15123

**Published:** 2015-10-12

**Authors:** Ming-Yen Lu, Ming-Pei Lu, Shuen-Jium You, Chieh-Wei Chen, Ying-Jhe Wang

**Affiliations:** 1 Graduate Institute of Opto-Mechatronics, National Chung Cheng University, Chia-Yi 62102, Taiwan; 2 Advanced Institute of Manufacturing with High-Tech Innovations, National Chung Cheng University, Chia-Yi 62102, Taiwan; 3 National Nano Device Laboratories, Hsinchu 300, Taiwan

## Abstract

In this study we measured the degrees to which the Schottky barrier heights (SBHs) are lowered in ZnO nanowire (NW) devices under illumination with UV light. We measured the I–V characteristics of ZnO nanowire devices to confirm that ZnO is an n-type semiconductor and that the on/off ratio is approximately 10^4^. From temperature-dependent I–V measurements we obtained a SBH of 0.661 eV for a ZnO NW Schottky device in the dark. The photosensitivity of Schottky devices under UV illumination at a power density of 3 μW/cm^2^ was 9186%. Variations in the SBH account for the superior characteristics of n-type Schottky devices under illumination with UV light. The SBH variations were due to the coupled mechanism of adsorption and desorption of O_2_ and the increase in the carrier density. Furthermore, through temperature-dependent I–V measurements, we determined the SBHs in the dark and under illumination with UV light at power densities of 0.5, 1, 2, and 3 μW/cm^2^ to be 0.661, 0.216, 0.178, 0.125, and 0.068 eV, respectively. These findings should be applicable in the design of highly sensitive nanoscale optoelectronic devices.

Great effort has been exerted over the past two decades in exploring the potential applications of nanostructures [e.g., nanowires (NWs), nanorods, nanowalls, nanobelts], taking advantage of their large surface-to-volume ratios and distinctive physical properties relative to those of their bulk materials. Among semiconductor nanomaterials, ZnO is one of most promising, having been proposed for a wide range of applications in optoelectronic devices^1^,^2^, energy harvesting devices^3^,^4^, optoelectronic memories^5^, field effect transistors^6^, and sensors^7^.

In our modern societies we are often exposed to ultraviolet (UV) radiation, from sunlight, artificial light sources, and electrical appliances. Although it is invisible to the human eye, UV light can cause skin disease and do much harm to our eyes and other parts of our bodies. Consequently, photon sensors/detectors are being developed for environmental monitoring in the UV spectral range, as well as for applications in flame detectors and signal receivers for optical communication^8^.

For practical use, UV detectors should exhibit excellent operational characteristics, including rapid response, high sensitivity, and good wavelength selectivity. Among them, nanodevices that can vary their electrical signals under illumination have attracted much attention because of their high sensitivity and ease of signal reading^9^,^10^,^11^. Such detectors can be categorized as either Schottky or ohmic nanodevices. Schottky nanodevices can display outstanding photon detection ability^12^,^13^ when the Schottky barrier height (SBH) is lowered after irradiating with light from an external source. This detection concept can also be adopted for the detection of molecules^14^ and gases^15^. Nevertheless, the correlation between the power of the illuminating light and the variation of the SBH has not been described previously. In this paper, we report our study into the mechanism of the photoresponse of ZnO NW Schottky devices. We used temperature-dependent I–V measurements to extract the SBHs of Schottky devices, as well as the variations in SBHs under different UV illumination power densities.

## Results

**Figure 1:**
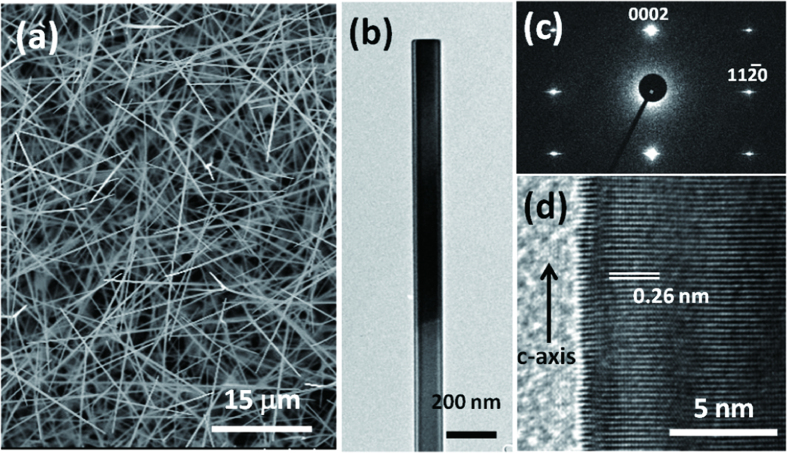
(**a**) SEM image of ZnO NWs grown on a Si substrate. (**b)** Low-magnification TEM image of a single ZnO NW. (**c**) SAED pattern of a single ZnO NW. (**d**) High-resolution TEM image of a single ZnO NW.

We grew the ZnO NWs through the vapor–liquid–solid (VLS) mechanism using a high-temperature vacuum furnace. The field emission scanning electron microscopy (FESEM) image in Fig. 1(a) reveals uniform ZnO NWs having lengths and diameters of approximately 70–80 μm and 100 nm, respectively. Fig. 1(b) presents a low-magnification transmission electron microscopy (TEM) image of a single ZnO NW; the selective-area electron diffraction (SAED) pattern in Fig. 1(c) confirms the single-crystalline nature of the NW, with diffraction spots corresponding to wurtzite-structured ZnO. In addition, the lattice spacing of 0.26 nm in the high-resolution TEM (HRTEM) image in Fig. 1(d) corresponds to the (0002) plane, also known as the c-axis, of ZnO.

**Figure 2:**
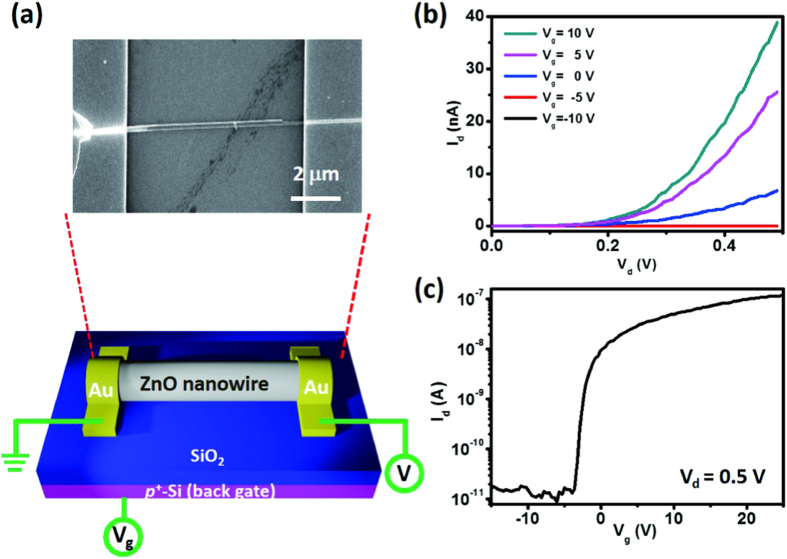
(**a**) Schematic representation of the ZnO NW device; inset: SEM image of a single–ZnO NW device. (The figure was drawn by Shuen-Jium You) (**b**) I_d_–V_d_ curves measured at various gate voltages. (**c**) I_d_–V_g_ curve recorded at a value of V_d_ of 0.5 V.

We used e-beam lithography and the lift-off process to fabricate the single–ZnO NW devices illustrated in Fig. 2(a); the inset presents a scanning electron microscopy (SEM) image of such a device. We selected Au for the electrodes of our ZnO NW Schottky devices, and a 300-nm SiO_2_/Si substrate as the back gate for measurement of field-effect transistor (FET) characteristics. Figure 2(b) displays drain current–drain voltage (I_d_–V_d_) curves plotted at various gate voltages (V_g_); the slope of each curve increased notably upon increasing the gate voltage from –10 to +10 V, with evidence for the channel beginning to be depleted at a value of V_g_ of approximately −5 V, suggesting that the ZnO NW device was an n-type FET with Schottky contacted characteristics. The sweeping I_d_–V_g_ characteristics of the FET at a value of V_d_ of 0.5 V are presented on a logarithmic scale in Fig. 2(c); the threshold voltage was –3.5 V, while the on/off ratio, approximately 10^4^, is comparable with those described in the literature for back-gate FETs^16^. The gate voltages applied to the devices were used to identify the conducting type and the FET performance of the ZnO NW; no gate voltages were applied to the devices in our subsequent experiments. We used the cylinder-on-plate model to estimate the mobility and the carrier concentration of the ZnO NW in an ohmic-contacted ZnO NW device (Fig. S1)^17^, which had a length of 10.3 μm, a diameter of 120 nm, and a SiO_2_ gate oxide layer that was 300 nm thick; the mobility and the carrier concentration of the ZnO NW were 25–33 cm^2^/V**·**s and (1–3) × 10^16^ cm^**–**3^, respectively.

**Figure 3:**
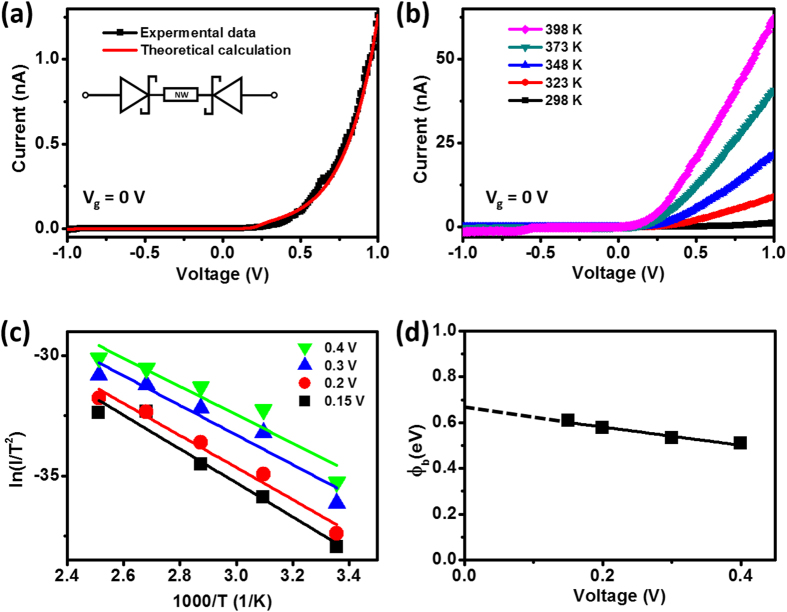
(**a**) I–V curve (black) and fitted data (red) of a ZnO NW Schottky device. The inset shows the circuitry of the back-to-back Schottky device. (**b**) I–V curves of the Schottky device measured at temperatures ranging from 298 to 398 K. (**c**) Plots of ln(I/T^2^) versus 1000/T at various applied voltages; the data had been extracted from (**b**). (**d**) Values of φ_b_ plotted with respect to the applied voltage.

The I–V curves of the Schottky-contacted ZnO NW devices operated in the dark [Fig. 3(a), black line] suggested that they possessed typical rectifier characteristics. In terms of the device with single Schottky contact, the device at reverse bias is known as the off-state (The carrier flow from metal to semiconductor), whereas, the device at forward bias is at on-state (the carrier flow from semiconductor to metal) for the Schottky device operation. The back-to-back Schottky device is composed of two Schottky contacts in series, the simplified circuitry of the device is illustrated in the inset of Fig. 3(a). The current passing through the device always confronts two Schottky contacts with different states, on-state and off-state, respectively. The resistance at off-state is relatively higher than that at on-state, therefore, the off-state current dominates the current flow in back-to-back Schottky device, that is to say, the current measured from back-to-back Schottky device should be at off-state under any circumstances. On the other hand, for the device with the same SBHs at two ends, the I–V curve is symmetric, whereas, the asymmetric I–V characteristic presented in this work indicates the disparate SBHs at two ends of the devices, the band diagrams and the simulated I–V curves of abovementioned conditions are illustrated in Figs S2(a) and S2(b), respectively. The rectifier characteristics of a Schottky device can be expressed physically through thermionic emission theory; thus, the values of the SBHs (φ_b_) of devices can be determined using various techniques^18^,^19^. For example, Chiquito *et al.* proposed a general equation to describe the behavior of current flow across a back-to-back Schottky-contacted device^20^, in turn allowing determination of its various parameters (e.g., φ_b_ and ideality factors). We used their model to fit our I–V data; the resulting curve [Fig. 3(a), red line] fits the data well, allowing us to extract values of φ_b_ of 0.68 and 0.75 eV for the magnitudes of the SBHs at the two contacts of the ZnO NW device, respectively. The presence of such uneven values for the barrier heights in our device gave rise to the asymmetrical I–V characteristics. The leakage current of our measurement system was a few picoamperes during the temperature-dependent I–V measurements, comparable with the current level at negative applied voltage; accordingly, we focused our attention on the temperature-dependent I–V measurements under the positive applied voltage regime for extraction of the barrier height. From the viewpoint of device physics, SBHs can be extracted based on the thermionic emission theory via temperature-dependent I–V measurements, the equation describing the current across the Schottky contact is^21^


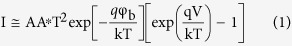


where A is the contact area; A* is the Richardson constant; T is the absolute temperature; φ_b_ is the effective SBH; and V, q, and k are the bias applied to the device, unit electronic charge, and the Boltzmann constant, respectively. More importantly, several literature reports suggest that the voltage-dependent features of the SBH can be observed in nanodevices^20^,^22^,^23^,^24^. For the two-terminal Schottky devices, the voltage applied between electrodes brings the electrical field in between, and then causing the voltage-dependent behavior of the SBHs due to the image charge lowering^25^; thus, the ability to eliminate the influence of the applied voltage on the extracted SBHs is an important issue for extracting the barrier heights. Accordingly, we adopted a method of using the linear extrapolation proposed by J. R. Chen *et al.* to extract the ideal barrier heights of Schottky devices^24^. The I–V characteristics of our Schottky devices were temperature-dependent, with the measured currents of the devices increasing upon increasing the temperature from 298 to 398 K [Fig. 3(b)]. Subsequently, we performed the following analysis to eliminate the influence of temperature and applied voltages when determining the barrier height. Eq. 1 can be transformed into the Arrhenius equation


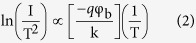


Figure 3(c) displays the Arrhenius plots of I/T^2^ with respect to 1000/T at applied voltages of 0.15, 0.2, 0.3, and 0.4 V. We extracted values of φ_b_ from the slopes of these linear plots for each applied bias. Figure 3(d) presents these values plotted with respect to the SBH barrier height (φ_b0_)—that is, a value of 0.661 eV for the ZnO NW Schottky device operated in the dark, a value that differs by only 2.8% from that calculated from the model proposed by Chiquito *et al.* The extraction analysis of values of φ_b0_ from temperature-dependent I–V measurements can also be adapted to other semiconductors^13^,^24^.

**Figure 4:**
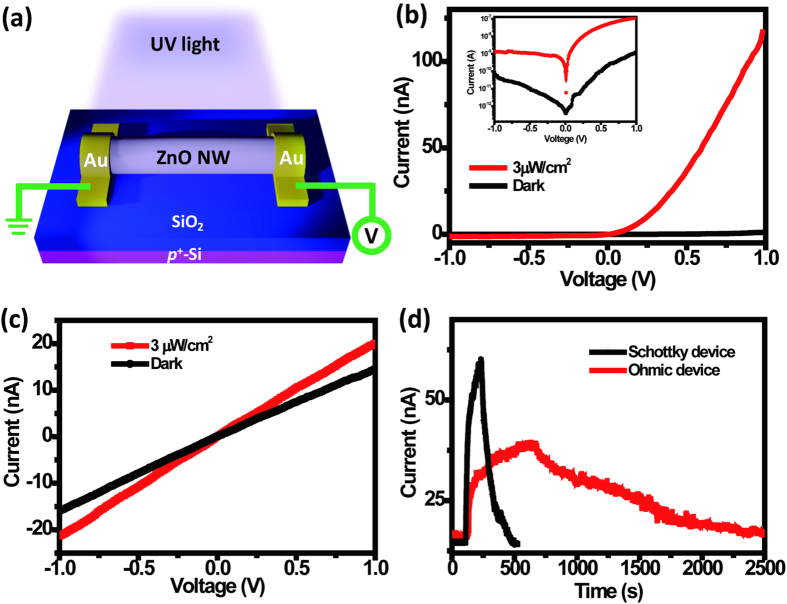
(**a**) Schematic representation of a ZnO device illuminated with UV light. (The figure was drawn by Shuen-Jium You) (**b**) I–V characteristics of a Schottky device in the dark (black) and under 365-nm UV light (red); inset: the same data plotted on a logarithmic scale. (**c**) I–V characteristics of an ohmic device in the dark (black) and under 365-nm UV light (red). (**d**) Photoresponses of the ZnO NW Schottky (black) and ohmic (red) devices under illumination with UV light at a power density of 1 μW/cm^2^.

Next, we measured the electrical properties of our ZnO NW Schottky devices under illumination with UV light. Figure 4(a) provides a cartoon representation of the setup for the photoresponse measurements. We used a 365-nm LED with tunable power as our UV light source. Figure 4(b) displays the I–V characteristics of our ZnO NW Schottky devices in the dark (black line) and under illumination with UV light at 3 μW/cm^2^ (red line); the curves reveal not only rectifier characteristics but also a high response toward the UV light (the inset presents the results on a logarithmic scale). The sensitivity of the photoresponse is defined herein as


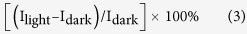


We estimated a sensitivity of 9186% for the ZnO NW Schottky device at an applied voltage of 1 V. For comparison, we also fabricated ZnO NW ohmic devices by depositing Pt:Ga metal onto the NW using a focused ion beam. Regardless of the band alignments between Pt and semiconductors, a high-intensity Ga beam can modify the contact area of an NW surface and lower the barrier height to achieve ohmic contact in a device^26^,^27^. Figure 4(c) presents the I–V characteristics of our ZnO NW ohmic device in the dark and under illumination with UV light; the sensitivity of this ZnO NW ohmic device toward the UV light was only 37.9%. Thus, the sensitivity of our Schottky device was approximately 240 times greater than that of our ohmic device. Meanwhile, we also used the responsivity to examine the performance of the device under UV illumination; it is defined as


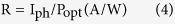


where I_ph_ is the difference between the measured currents in the dark state and under UV illumination, and P_opt_ is the incident power on the NW (power density of UV light × length × diameter of NW). For the Schottky device, the value of I_ph_ was 115 × 10^–9^ A, while the channel length and diameter of the ZnO were 20 μm and 120 nm, respectively. The value of R was approximately 1.6 × 10^6^ A/W at 1 V; for the ohmic device, it was calculated to be 7.6 × 10^4^ A/W. The high sensitivity and responsivity of Schottky devices have been attributed to their barrier lowering under UV illumination. Furthermore, Fig. 4(d) displays the photoresponses of our ohmic and Schottky ZnO NW devices under illumination with UV light. The enhanced conductivity of ZnO NW ohmic devices under UV illumination has been attributed to the adsorption and desorption of O_2_ on the NW surface^28^; because these processes are relatively slow, the photoresponses of ohmic devices toward UV light are also relatively slow. Nevertheless, our Schottky device exhibited higher sensitivity and faster responses and recovery times toward UV light than did our ohmic device. The rapid UV responses of Schottky ZnO NW devices are associated with lowering of their SBHs under UV illumination^7^,^12^. The barrier lowering of Schottky devices under UV illumination arises mainly through two mechanisms: the adsorption and desorption of O_2_ and the increase in carrier density. Several studies have revealed that Schottky barriers are very sensitive to O_2_^15^,^29^. The O_2_ that adsorbed onto the NW surface captured an electron, forming a negatively charged oxygen ion [O_2_ + e^−^ → O_2_^−^] in the dark state. Once UV light illuminated the sample, the electron/hole pairs that were generated could be separated by the electric field at the depletion region. The photogenerated holes discharged negatively charged oxygen ions at the interface [O_2_^−^ + h^+^ → O_2_]; these processes modify the effective density of charge states at the interface^30^. Moreover, the photogenerated electrons migrate toward the channel and increase the carrier density of the NW^31^. Both of these mechanisms result in a decrease in the depletion width, with the voltage drop between the metal and the semiconductor (the applied voltage) within the interface region remaining approximately constant; as a result, the electrical field in the depletion region increased. The lowering of the Schottky barrier would occur as a result of the image charge lowering effect. The relationship between electrical field and Schottky barrier lowering (Δφ) is expressed as,


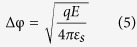


where q, E, and ε_S_ are the charge, the electric field, and the permittivity in the semiconductor, respectively. The I–V curves of the Schottky device under high-power-density UV illumination reveal that the rectifying behavior, due to the barrier height, become less apparent (Fig. S3). It is evident that UV illumination results in barrier-lowering in ZnO NW Schottky devices.

**Figure 5:**
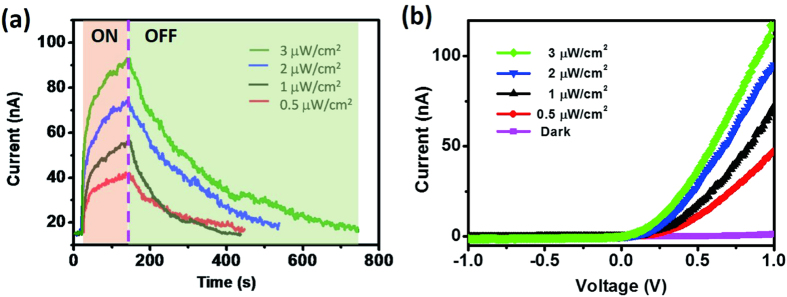
(**a**) Photoresponse of a ZnO Schottky device measured under illumination with UV light at power densities from 0.5 to 3 μW/cm^2^. (**b**) I–V curves of the Schottky device in the dark and under illumination with UV light (365 nm) at power densities from 0.5 to 3 μW/cm^2^.

Figure 5(a) plots the photoresponse of our Schottky device with respect to the power density of UV light; the sensitivity of our device was nearly linear with respect to the power density, while the response time was independent of it. Nevertheless, it took longer for the response to recover to its original state when illuminating with UV light at a higher power density. The power density of UV light used in this study was much lower than that of the UV light coming from the sun (a few milliwatts per square centimeter). According to the working principle, as long as electron/hole pairs could be generated, we would detect the UV light through a photoresponse; thus, such devices should be capable of detecting UV light in the environment. Figure 5(b) presents I–V curves of our Schottky device measured in the dark and under UV light (365 nm) at power densities ranging from 0.5 to 3 μW/cm^2^. The photocurrent increased and the turn-on voltage decreased upon increasing the power density of the incident light. The decrease in the turn-on voltage implied modulation of the SBH. The rapid responses and high sensitivities of Schottky devices toward illuminating light are also known to result from lowering of their SBHs^7^,^13^. To determine the photoresponse mechanism for our ZnO NW Schottky device, we performed a systematic study to extract the SBH variations of a device under illumination with UV light, using the abovementioned SBH extraction technique. The temperature-dependent I–V measurements under UV illumination at power densities of 0.5, 1, 2, and 3 μW/cm^2^ revealed (Figs S4 and S5) that, regardless of the illumination power density, the current flowing across the barrier increased upon increasing the temperature.

**Figure 6:**
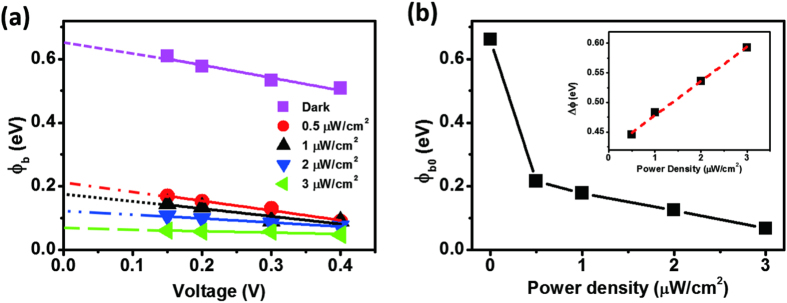
(**a**) Plot of the values of φ_b_ with respect to applied voltage under illumination with UV light at various power densities. (**b**) Values of φ_b0_ plotted with respect to the illumination power density; data were obtained from (**a**); inset: plot of Δφ_b0_ (the difference between the values of φ_b0_ under UV light and in the dark) with respect to the power density of the UV light.

Figure 6(a) displays plots of the values of φ_b_, determined from the temperature-dependent I–V measurements, with respect to the applied voltage. By extrapolating each line to the y-axis, we calculated SBHs in the dark and under UV illumination at power densities of 0.5, 1, 2, and 3 μW/cm^2^ of 0.661, 0.216, 0.178, 0.125, and 0.068 eV, respectively. The plot of the variations in SBH [Fig. 6(b)] features a dramatic drop once the device had been illuminated, implying that the SBHs were very sensitive to UV light. When the energy of the illuminating light is larger than the bandgap of ZnO, electron/hole pairs are generated in the ZnO; the photogenerated holes are involved in the desorption of O_2_ near the ZnO–Au interface, resulting in an increase in the majority carrier density, a lowering of the SBH, and a decrease in the depletion width. The inset to Fig. 6(b) displays the difference between the extracted SBH under UV illumination at a particular power density and the SBH in the dark state (φ); the value of φ increased upon increasing the illumination power density, suggesting that the lowering of the SBH was related directly to the illumination power density, consistent with the mechanism described above for Schottky photodetectors.

## Discussion

We have synthesized uniform ZnO NWs through the VLS growth mechanism in a high-temperature furnace. Through TEM analysis, we characterized these ZnO NWs as having grown along the c-axis. The I–V characteristics of our ZnO NW device revealed that the ZnO was an n-type semiconductor having an on/off ratio of approximately 10^4^. Through temperature-dependent I–V measurements, we determined the SBH of the ZnO NW Schottky device in the dark to be 0.661 eV. The photosensitivity of the Schottky device was 9186% under UV illumination at 3 μW/cm^2^, more than 240 times greater than that of a corresponding ohmic device. We attribute the significant differences in sensitivity and response of the ohmic and Schottky devices toward UV light to their different response mechanisms. For an n-type ohmic device, the desorption and adsorption of O_2_ on the NW surface have a major effect on its response; in contrast, variations in the SBH are responsible for the superior characteristics of an n-type Schottky device. The SBH variations were due to the coupled mechanism of adsorption and desorption of O_2_ and the increase in carrier density. Furthermore, from temperature-dependent I–V measurements, we determined the variations in SBH in the dark and under UV light at power densities of 0.5, 1, 2, and 3 μW/cm^2^ to be 0.661, 0.216, 0.178, 0.125, and 0.068 eV, respectively. The difference between the SBH under UV illumination and that in the dark exhibited a linear relationship with respect to the power density of the UV light.

## Methods

ZnO NWs were synthesized through the vapor transport growth method (VLS mechanism), using 5-nm-thick Au films as catalysts, in a high-temperature vacuum furnace, as described previously^24^. A mixture of ZnO and C powders (equal weight) was used as the source and placed upstream in the high-temperature furnace; (100) Si substrates coated with 5-nm-thick Au films were placed downstream. Ar (140 sccm) and O_2_ (50 sccm) were used as carrier gases; the pressure was controlled at 4 torr. The furnace was heated to 1000 °C at a heating rate of 15 °C/min, maintained at 1000 °C for 30 min, and then cooled naturally to room temperature.

The morphologies and crystal structures of the ZnO NWs were characterized using FESEM (Hitachi S4800-I) and TEM (JEOL JEM-2010). The single–ZnO NW devices were fabricated through e-beam lithography; the ZnO NWs were first dispersed on a 300-nm SiO_2_/Si substrate and then subjected to lithography, metal electrode deposition, and lift-off processes; Au was deposited to form electrodes and achieve the Schottky devices. ZnO NW devices with ohmic contacts were also fabricated using an FIB system. The electrical characteristics of single–ZnO NW devices were measured using a semiconductor characterization system (Keithley 2636B); for temperature-dependent I–V and photoresponse measurements of the ZnO NW devices, this system was combined with a hot stage and a home-built power-controllable UV light illumination system.

## Additional Information

**How to cite this article**: Lu, M.-Y. *et al.* Quantifying the barrier lowering of ZnO Schottky nanodevices under UV light. *Sci. Rep.* **5**, 15123; doi: 10.1038/srep15123 (2015).

## Supplementary Material

Supplementary Information
