# Supplementary material for: Quantifying the barrier lowering of ZnO Schottky nanodevices under UV light
Source: Sci Rep. 2015 Oct 12;5:15123. doi: 10.1038/srep15123 (PMC4601028; doi:10.1038/srep15123)
Supplement: Supplementary Information [file srep15123-s1.pdf]

Supplementary Information

Quantifying the barrier lowering of ZnO Schottky nanodevices under UV light

Ming-Yen Lu[1,2], Ming-Pei Lu[3], Shuen-Jium You[1], Chieh-Wei Chen[1], and Ying-Jhe Wang[1]

[1] Graduate Institute of Opto-Mechatronics, National Chung Cheng University, Chia-yi 62102, Taiwan

[2] Advanced Institute of Manufacturing with High-Tech Innovations, National Chung Cheng University, Chia-Yi 62102, Taiwan

[3] National Nano Device Laboratories, Hsinchu 300, Taiwan

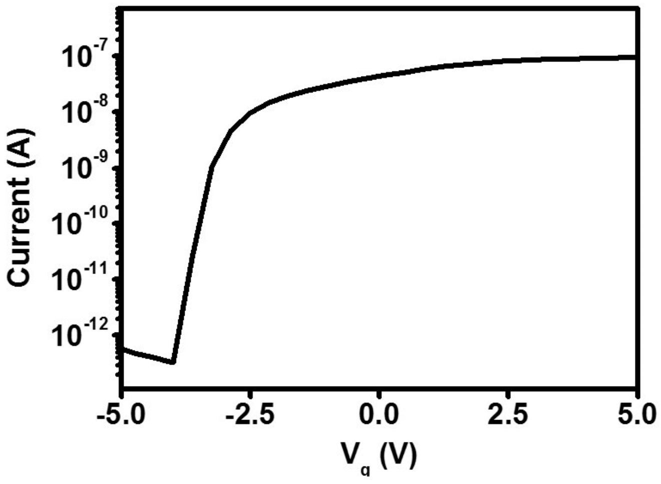

Figure S1. I_d–V_g curve of ZnO NW ohmic device recorded at a value of V_d of 1V

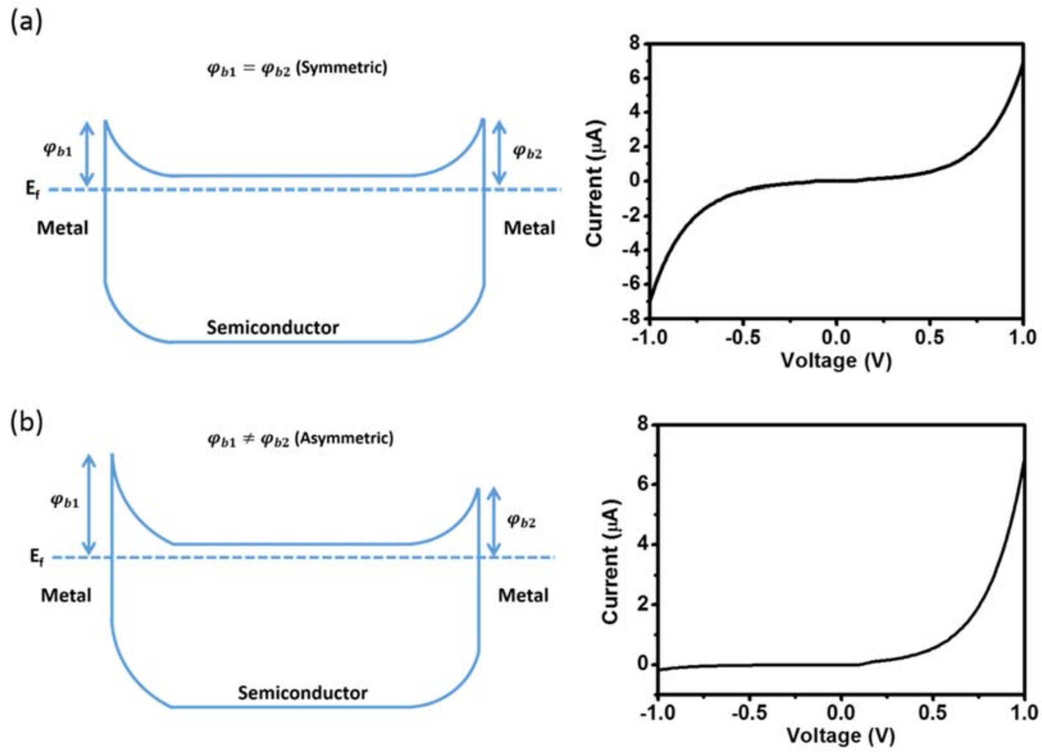

Figure S2. The band diagrams of the back-to-back Schottky device with (a) the same SBHs and (b) uneven SBHs. The corresponding simulated I-V curves are symmetric and asymmetric, respectively.

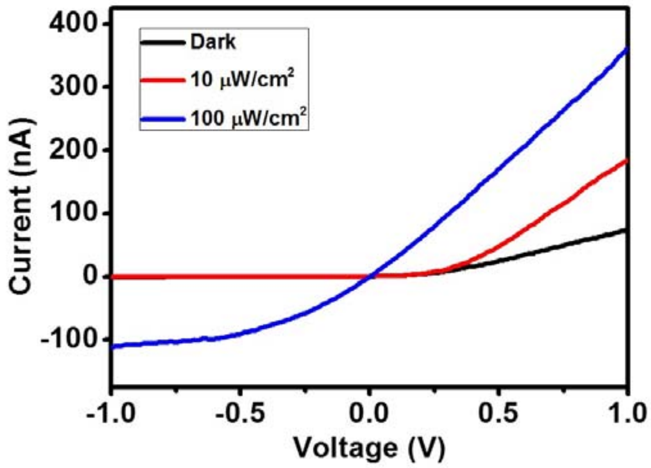

Figure S3. I-V curves of ZnO NW Schottky device at dark state and under 10 and 100 μW/cm^2 UV light illuminations.

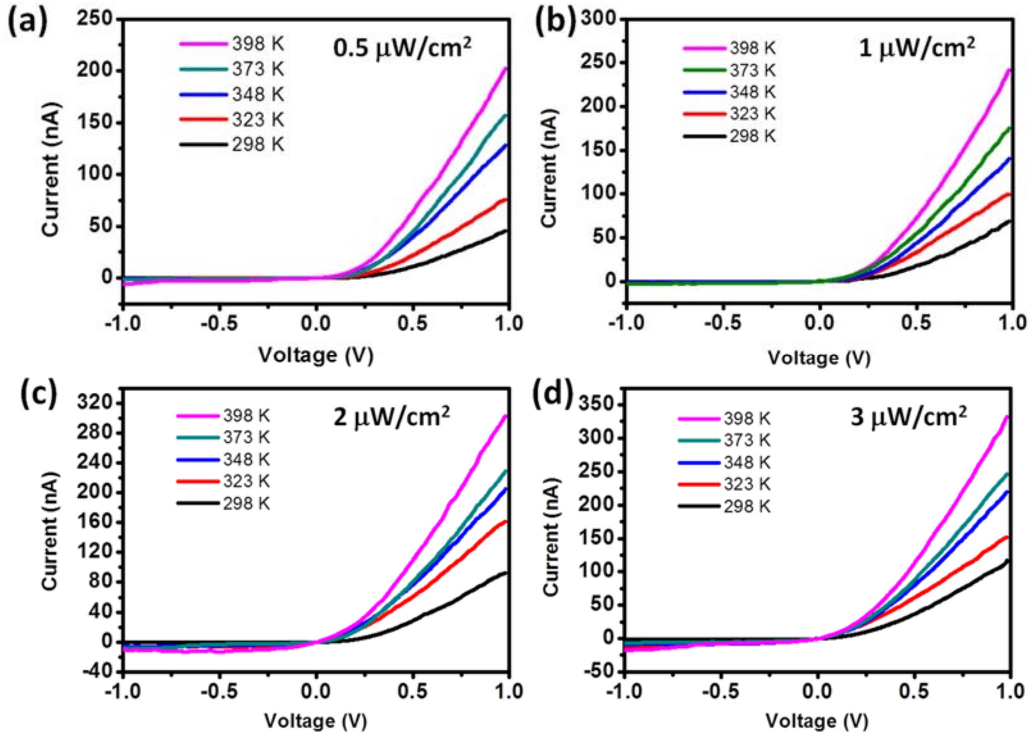

Figure S4. *I*–*V* curves of the Schottky device measured at temperatures ranging from 298 to 398 K under different UV illumination power density, (a) 0.5 μm/cm^2, (b) 1 μm/cm^2, (c) 2 μm/cm^2, and (d) 3 μm/cm^2.

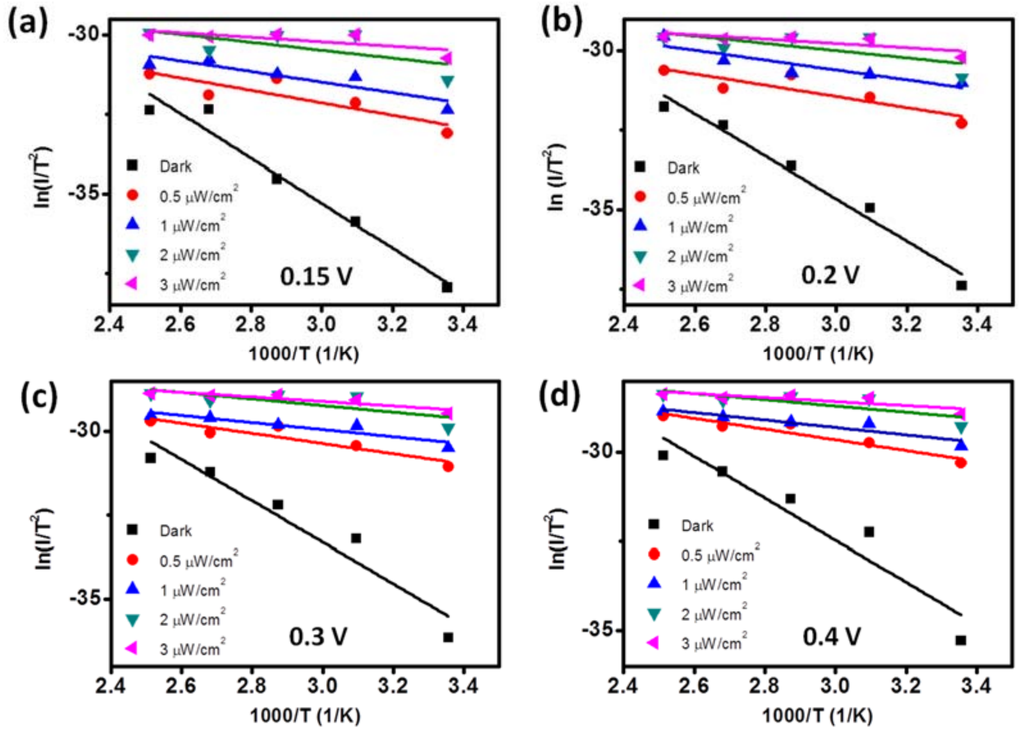

Figure S5. Plots of $\ln(I/T^2)$ versus $1000/T$ of devices measured under different UV illuminated power density, the data are extracted from Fig. S1 at (a) V = 0.15 V, (b) V= 0.2 V, (c) V=0.3 V, and (d) V=0.4 V.
